# Safety of Tubal Occlusion by Minilaparotomy Provided by Trained Clinical Officers Versus Assistant Medical Officers in Tanzania: A Randomized, Controlled, Noninferiority Trial

**DOI:** 10.9745/GHSP-D-18-00108

**Published:** 2018-10-03

**Authors:** Mark A. Barone, Zuhura Mbuguni, Japhet Ominde Achola, Annette Almeida, Carmela Cordero, Joseph Kanama, Adriana Marquina, Projestine Muganyizi, Jamilla Mwanga, Daniel Ouma, Caitlin Shannon, Leopold Tibyehabwa

**Affiliations:** a EngenderHealth, New York, NY, USA. Now with Population Council, New York, NY, USA.; b Tanzania Ministry of Health, Community Development, Gender, Elderly and Children, Dar es Salaam, Tanzania.; c EngenderHealth, Nairobi, Kenya.; d RESPOND Tanzania Project, EngenderHealth, Dar es Salaam, Tanzania. Now with Jhpiego, Dar es Salaam, Tanzania.; e EngenderHealth, New York, NY, USA.; f RESPOND Tanzania Project, EngenderHealth, Dar es Salaam, Tanzania.; g Association of Gynaecologists and Obstetricians of Tanzania, Dar es Salaam, Tanzania.; h EngenderHealth, Nairobi, Kenya. Now with Population Council, Nairobi, Kenya.

## Abstract

**Background:**

Tubal occlusion by minilaparotomy is a safe, highly effective, and permanent way to limit childbearing. We aimed to establish whether the safety of the procedure provided by trained clinical officers (COs) was not inferior to the safety when provided by trained assistant medical officers (AMOs), as measured by major adverse event (AE) rates.

**Methods:**

In this randomized, controlled, open-label noninferiority trial, we enrolled participants at 7 health facilities in Arusha region, Tanzania, as well as during outreach activities conducted in Arusha and neighboring regions. Consenting, eligible participants were randomly allocated by a research assistant at each site to minilaparotomy performed by a trained CO or by a trained AMO, in a 1:1 ratio. We asked participants to return at 3, 7, and 42 days postsurgery. The primary outcome was the rate of major AEs following minilaparotomy performed by COs versus AMOs, during the procedure and through 42 days follow-up. The noninferiority margin was 2%. The trial is registered with ClinicalTrials.gov, Identifier NCT02944149.

**Results:**

We randomly allocated 1,970 participants between December 2016 and June 2017, 984 to the CO group and 986 to the AMO group. Most (87%) minilaparotomies were conducted during outreach services. In the intent-to-treat analysis, 0 of 978 participants had a major AE in the CO group compared with 1 (0.1%) of 984 in the AMO group (risk difference: –0.1% [95% confidence interval: –0.3% to 0.1%]), meeting the criteria for noninferiority. We saw no evidence of differences in measures of procedure performance, participant satisfaction, or provider self-efficacy between the groups.

**Conclusions:**

Tubal occlusion by minilaparotomy performed by trained COs is safe, effective, and acceptable to women, and the procedure can be safely and effectively provided in outreach settings. Our results provide evidence to support policy change in resource-limited settings to allow task shifting of minilaparotomy to properly trained and supported COs, increasing access to female sterilization and helping to meet the rising demand for the procedure among women wanting to avoid pregnancy. They also suggest high demand for these services in Tanzania, given the large number of women recruited in a relatively short time period.

## Abstract (precis)

Trained clinical officers—nonphysicians with 3 years of specialized training—conducted the procedure safely and effectively compared with procedures performed by more advanced assistant medical officers. This evidence supports policy change allowing properly trained and supported clinical officers to perform minilaparotomy.

## INTRODUCTION

Globally, modern contraceptive use has risen substantially over the last 45 years from 36% in 1970 to 64% in 2015,^1^ yet unmet need for family planning remains high. In 2017, an estimated 214 million women of reproductive age living in developing regions of the world wanted to avoid pregnancy but were not using a modern contraceptive method, accounting for 84% of unintended pregnancies in these regions.^2^ In many cases, this leads to a high burden of maternal and child morbidity and mortality and to unsafe abortions.^2^ Unmet need is highest in sub-Saharan Africa, and although the number of women wanting to limit future childbearing in this region has been rising, many of these women use less effective short-acting methods of contraception instead of more effective methods such as female sterilization.^3^^,^^4^

Tubal occlusion via minilaparotomy, using local anesthesia and analgesia, with or without systemic sedation, is the simplest way to provide female sterilization. The surgery is minor and can be performed in resource-limited settings on an outpatient basis, with low risk of complications.^5^^,^^6^ This procedure can be performed anytime that pregnancy can be ruled out (commonly referred to as “interval” sterilization), or within the first 7 days following vaginal delivery or first-trimester abortion; it is not recommended between 8 and 42 days postpartum but could be performed anytime thereafter.^7^

One reason women may not use female sterilization is limited access to services. In Tanzania, for example, the Ministry of Health, Community Development, Gender, Elderly, and Children (MOHCDGEC) recognizes that most health facilities are understaffed, more so in rural areas, and that a shortage of trained providers affects the availability of health care services, including female sterilization.^8^ Family planning providers themselves report that service provision is hampered by a mismatch between what clients want and what facility staff are able to provide or what certain cadres are *allowed* to provide under current government regulations.^9^ Access to health services can be expanded with task shifting, the delegation of some tasks to less-specialized health workers. We use the term “task shifting” to mean situations where a less-specialized health worker conducts the entire procedure (e.g., a surgical procedure) on his or her own (some may refer to this as task sharing).^10^ Task shifting of surgical procedures to mid-level providers has improved access to lifesaving interventions, with clinical officers (COs) and nurses demonstrating outcomes similar to those of their higher-level counterparts in Malawi,^11^ Mozambique,^12^^–^^14^ and Tanzania.^15^

One reason women may not use female sterilization is limited access to services.

Task shifting could increase access to tubal occlusion, especially in rural areas where demand for family planning is high and where most health services are provided by nonphysicians.^16^^,^^17^ In fact, World Health Organization (WHO) guidelines include COs among those considered competent to provide tubal occlusion. Although the guidance panel accepted that the procedure was within the COs' competency, the panel members did not review the available evidence to support their recommendation.^18^ Two systematic reviews, which included older studies conducted primarily in the 1970s and 1980s, were published after the WHO guidelines were released.^19^^,^^20^ Results of both reviews suggest that task shifting of tubal occlusion to nonphysicians may be a safe and effective approach to increasing contraceptive access.

Task shifting provision of tubal ligation to mid-level providers could increase access to the procedure, especially in rural areas.

Three, more recent nonrandomized studies offer additional support on the safety of task shifting tubal occlusion by minilaparotomy.^21^^–^^23^ No major adverse events (AEs), defined by the authors as complications serious enough to require referral to a hospital, were reported among 164 women in Malawi through 14 days of follow-up after minilaparotomy performed by COs.^21^ In Uganda, a major AE rate of 1.5% was reported among 518 women following minilaparotomy performed by a CO through 45 days after surgery.^22^ The authors defined major AEs as events causing long-term incapacity or disability *and* requiring hospitalization, as well as failed minilaparotomy procedures. Finally, in Ethiopia, the rate of major AEs, defined by the authors as AEs requiring significant follow-up care or hospitalization, as well as failed procedures, among 276 women who had a minilaparotomy performed by a CO was 3%, with 6 of the 8 AEs being failure to complete the procedure.^23^

Overall, the available evidence is limited, and well-designed clinical trials are needed to definitively demonstrate the safety, efficacy, and acceptability of task shifting tubal occlusion to mid-level providers.^19^^–^^23^ We aimed to establish whether the safety of tubal occlusion by minilaparotomy provided by trained COs was not inferior to the safety of the procedure when provided by trained assistant medical officers (AMOs), as measured by major AE rates.

## METHODS

### Study Design and Participants

We conducted a randomized, controlled, open-label noninferiority trial comparing the safety of tubal occlusion by minilaparotomy when performed by trained COs and by trained AMOs at 7 study sites (2 district hospitals and 5 health centers) in Arusha region in northern Tanzania. We also recruited participants during outreach activities in Arusha, Dodoma, Kilimanjaro, Manyara, and Singida regions, since this approach is part of the MOHCDGEC's strategy to increase access to family planning. During outreach events, the trained COs and AMOs from the study sites traveled to and performed minilaparotomy procedures at other facilities where it was not routinely available.

**Figure:**
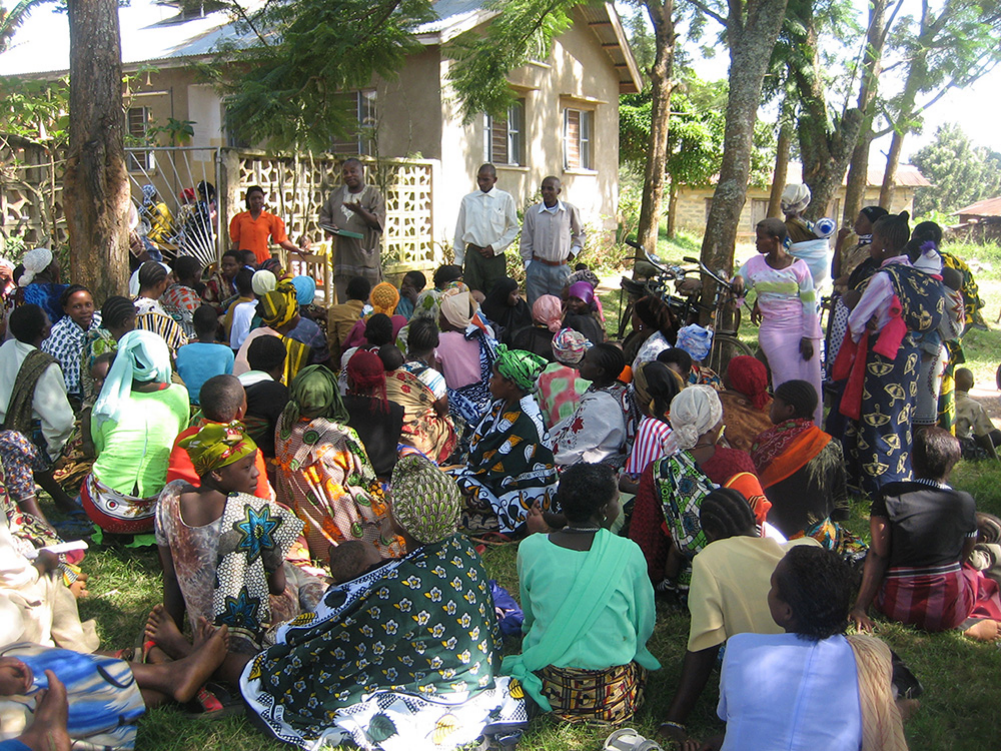
Health facility staff discuss family planning options with women waiting for outreach services in northern Tanzania. © 2016 EngenderHealth

We included women if they met the following inclusion criteria:

- were aged 18 years and older;
- requested and consented to tubal occlusion and provided informed consent for study participation;
- were of sound mind, in good general health, and deemed suitable to undergo tubal occlusion by minilaparotomy in accordance with the MOHCDGEC guidelines;
- understood study procedures and requirements;
- agreed to return for follow-up visits; and
- provided contact information.

We excluded women if they:

- were pregnant, based on the results of a rapid pregnancy test;
- were between 8 and 42 days postpartum or postabortion;
- had a known allergy or sensitivity to lidocaine or other local anesthetic;
- took medication contraindicating elective surgery;
- had previous abdominal or pelvic surgery;
- had a local skin infection near the area of the intended incision;
- had severe anemia, a coagulation disorder, hypertension, acute deep venous thrombosis, pulmonary embolism, or current ischemic heart disease;
- had unexplained vaginal bleeding, malignant gestational trophoblastic disease, cervical, endometrial, and/or ovarian cancer, pelvic inflammatory disease (within the last 3 months), or current purulent cervicitis, chlamydial infection, and/or gonorrhea;
- had current symptomatic gall bladder disease, active viral hepatitis, tuberculosis of pelvic organs, acute bronchitis or pneumonia, or systematic infection or gastroenteritis; or
- were currently participating in another biomedical research study.

The protocol was reviewed and approved by the National Institute for Medical Research, Tanzania, Dar es Salaam, and the Western Institutional Review Board, Puyallup, WA, USA.

### Randomization and Masking

Randomization was done using permuted blocks with randomly varying block sizes of 4 to 8 within each site. We randomized participants in a 1:1 ratio (i.e., minilaparotomy conducted by a CO or by an AMO). We concealed allocation through use of a text-message service (Sealed Envelope Ltd, London, UK, www.sealedenvelope.com). A researcher unaffiliated with the study computer-generated the random allocation sequence, which we then uploaded to Sealed Envelope before the start of recruitment. We randomized participants after screening had been conducted, a woman's eligibility for study participation had been confirmed, and just prior to start of the minilaparotomy procedure. Research assistants sent a text message requesting that a participant be randomized and received the random allocation in a text message reply. All study sites recruited participants until the total sample size had been reached. Because of the nature of the health facilities and services and the low availability of clinical staff at study sites, we were unable to mask participants, coinvestigators, those assessing outcomes, or other study staff to treatment allocation.

### Service Providers and Clinical Training

Minilaparotomies were conducted by 7 COs and 7 AMOs employed by the MOHCDGEC, with 1 CO and 1 AMO stationed at each study site. In Tanzania, COs are nonphysician health care providers who have undergone a standard 3-year training program. AMOs are COs who have at least 3 years of clinical work experience and who have completed an additional 2-year training program (Table 1).

**TABLE 1.** Background Characteristics of Service Providers Conducting Tubal Ligations in the Study

	Clinical Officers (n=7)	Assistant Medical Officers (n=7)
	3-year CO training course	≥3 years of CO clinical work, plus 2-year AMO training course
**Sex**		
Female	1	2
Male	6	5
**Age, years, median (range)**	29 (27, 57)	44 (36, 59)
**No. of years in career, median (range)**	3 (2, 31)	7 (2, 12)
**Type of facility** ^a^		
District hospital	2	3
Health center	5	6
Dispensary	1	1
Private hospital	1	0
No. with surgical experience before the minilaparotomy training	4	6
**Frequency performing surgery** ^b^		
Daily	0	1
Weekly (1–5/week)	2	4
Irregularly	2	1
**No. reporting experience with types of surgery** ^b^		
Abscess incision and drainage	1	3
Appendectomy	0	3
Cesarean delivery	0	6
Circumcision	3	0
Cyst excision	1	0
Hernia repair	0	1
Laparotomy for ruptured ectopic pregnancy	0	2
Lipoma removal	1	0
Wound repair	1	0

Abbreviations: AMO, assistant medical officer; CO, clinical officer.

a At current and previous postings; some worked at more than 1 type of facility during their career.

b Among those reporting surgical experience before the minilaparotomy training.

In Tanzania, clinical officers are nonphysician providers who have undergone a standard 3-year training program; assistant medical officers have an additional 3 years of clinical work experience and 2 more years of training.

The AMOs were older than the COs and more established in their careers, with only 2 of 7 AMOs having worked 7 years or less as an AMO compared with 6 of 7 COs having worked 7 years or less as a CO (with an outlier who had been a CO for 31 years). At the time of the study, 5 CO/AMO pairs were working at health centers and 2 at district hospitals. Several of the COs and AMOs had previously worked at other-level health facilities. Both the COs and AMOs provided a wide range of preventive, diagnostic, and treatment services as part of their clinical duties.

To ensure that all providers had adequate skills and used standardized procedures, prior to the start of the study we trained them to perform tubal ligation by minilaparotomy. None of the COs or AMOs had prior experience performing the procedure, although 4 of 7 COs and 6 of 7 AMOs had experience conducting other surgical procedures. The surgical experience of the COs was limited to minor surgeries such as drainage of abscesses or male circumcision, while the AMOs had experience conducting more complex surgery such as cesarean deliveries and appendectomies (Table 1). The 11-day competency-based training followed MOHCDGEC guidelines and standards.^24^ In keeping with national and international standards, we also included in the training surgical assistants who would assist the COs and AMOs while they performed the minilaparotomies.

The training included classroom sessions, practice with models, observation of minilaparotomy procedures, and conduct of procedures by the participants under supervision during the training workshop and post-training follow-up. Training covered applied anatomy, counseling, preoperative client assessment and preparation, pain management, emergency preparedness, minilaparotomy surgical skills, postsurgical assessment, follow-up, and complications prevention and management, as well as infection prevention practices relevant to minilaparotomy. We used pretests and posttests to assess individual trainees' change in knowledge. Providers used learning guides throughout the training, and trainers assessed the trainees' skills performance using an observation checklist in the training workshop and post-training follow-up. During the workshop, each provider conducted 5 procedures with assistance and coaching from a trainer. During post-training follow-up, the providers conducted minilaparotomy procedures at their work stations under a trainer's supervision. Although we do not have details on the number of procedures conducted under the trainer's supervision during post-training follow-up, all providers were deemed competent before the start of the study.

Other than during post-training follow-up, the providers were asked not to conduct any minilaparotomy procedures outside the context of the study, both before study recruitment began and once the study was underway.

### Procedures

After a research assistant obtained legally effective (signed or witnessed) informed consent, we evaluated each potential participant for clinical eligibility according to the study inclusion and exclusion criteria noted above. A research assistant then randomized eligible participants as described above, and in most cases minilaparotomy was performed on the same visit (or if not, within 7 days of screening). We asked women to void before the procedure and gave them injectable atropine and diclofenac preoperatively. Sedation is not included as part of pain management in the MOHCDGEC guidelines for minilaparotomy and was not used in the study.^24^ We performed all minilaparotomy procedures using 1% injectable lidocaine for local anesthesia and a uterine elevator, tubal hook and the modified Pomeroy technique for tubal occlusion, as per MOHCDGEC guidelines.^24^

**Figure:**
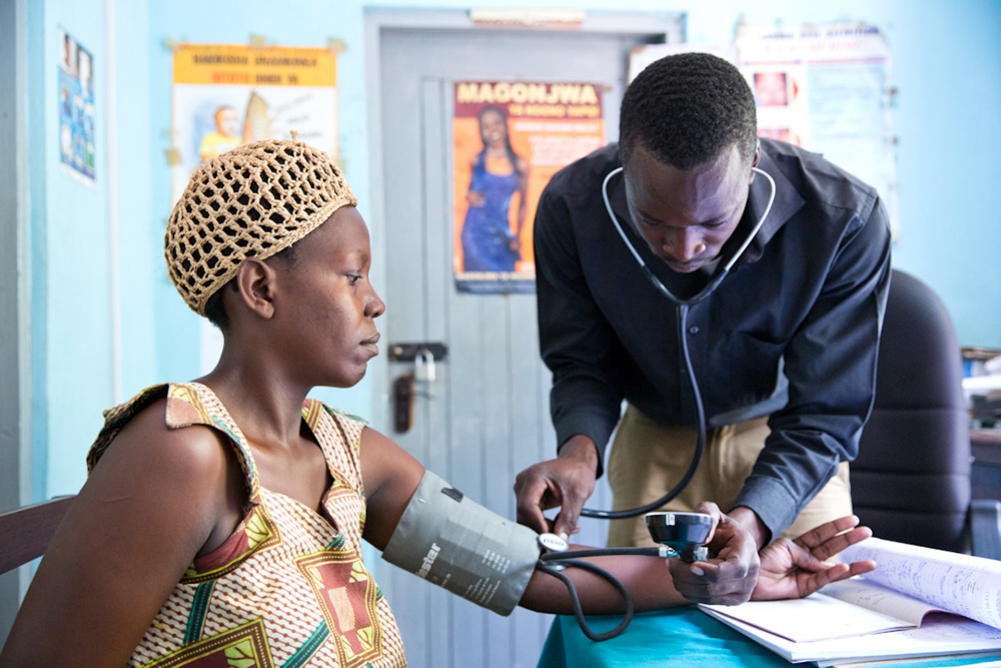
A clinical officer screens a woman for tubal ligation in a health facility in northern Tanzania. © 2014 EngenderHealth/S. Lewis

COs are not allowed by Tanzania government regulations to perform minilaparotomy; however, we received permission from the MOHCDGEC for the trained COs to perform minilaparotomies during the study as long as all procedures were under the supervision of a physician experienced with and qualified to perform minilaparotomy. Supervisors were present during all procedures conducted by both COs and AMOs, to ensure comparability between the 2 treatment groups. Supervisors were able to take over the procedure if necessary for the health and well-being of the participants or if the CO or AMO was unable to complete the procedure. They also were able to provide verbal instructions or assist a provider having difficulty with a procedure. Data were gathered on any assistance provided by supervisors.

After the minilaparotomy procedures, participants remained at the site for several hours, were monitored for any problems, and were given postoperative instructions before being discharged. We asked participants to return for 3 scheduled follow-up visits, at 3, 7, and 42 days postsurgery. We provided participants 5000 Tanzanian shillings (approximately US$2.25) to cover time and transport costs for each of the 3 scheduled follow-up visits. Providers scheduled additional visits as clinically necessary and informed participants that they should return to the site at any time if they had problems or concerns related to the procedure. During both scheduled and unscheduled follow-up visits, we gathered data on physical exam findings, AEs, and participants' experience and satisfaction postsurgery. Follow-up visits were conducted by available qualified providers; it was not practical to ensure that outcomes were assessed by someone other than the provider who had conducted the minilaparotomy. All medical procedures in the trial were conducted under the oversight of the MOHCDGEC.

### Outcomes

The primary outcome was safety, defined by the overall rate of major AEs (Box) following minilaparotomy performed by COs versus AMOs, during the procedure and through 42 days follow-up. All AEs were graded according to criteria defined before the start of the study. We defined minor AEs as any deviation from the normal postoperative course where treatment was limited to observation, conservative therapy (e.g., pressure to relieve bleeding or local wound care), or medication (e.g., antiemetics, antibiotics, or pain relievers).

BOX **Major Adverse Events**

1. Injuries to abdominal viscera, pelvic abscess, or severe peritonitis leading to unintended major surgery
2. Severe intra- or immediate postoperative hemorrhage requiring blood transfusion
3. Febrile morbidity (oral temperature greater than 38° C on at least 2 postoperative days, excluding the first 24 hours after surgery)
4. Life-threatening event (including cardiopulmonary crisis or anaphylaxis)
5. Readmission to the hospital any time after her discharge after the minilaparotomy through the end of follow-up due to a complication related to the minilaparotomy
6. Death or complication resulting in death occurring within 42 days of the surgery related to the minilaparotomy procedure

The primary outcome was safety, defined by the overall rate of major adverse events.

Prespecified secondary outcomes included:

- Rates of major and minor AEs following minilaparotomy procedures performed by COs vs. AMOs at different time points (i.e., intraoperatively, immediately postoperative, and at each follow-up visit)
- Differences in performance of minilaparotomy procedures between COs and AMOs (e.g., procedure times, requests for verbal instruction from the supervisor due to difficulty performing the procedure, requests for the supervisor to assist with the procedure, inability to complete the procedure, and maximum reported pain experienced by the participant during the procedure on a scale of 0=no pain to 10=worst possible pain)
- Participant satisfaction with the procedure performed by COs versus AMOs based on reported level of satisfaction (4-category ordinal scale: very satisfied to very dissatisfied)
- Provider self-efficacy, defined by providers' self-reported level of confidence, comfort, and perception of their ability to perform minilaparotomy

### Statistical Analysis

We assessed noninferiority of the safety of minilaparotomy provided by COs compared with AMOs in terms of the proportion of participants experiencing a major AE by Day 42 postsurgery, with a 2% predefined noninferiority margin chosen on the basis of a combination of experts' clinical judgment and statistical reasoning based on the results of previously reported AE rates following tubal occlusion by minilaparotomy.^25^ Assuming a 3% major AE rate in the control group (based on data from the previously reported studies), noninferiority would be shown within the margin of 2% at a 1-sided significance level of α=0.05 and a power of 80% (calculated when AE rates in both arms are the same) with a sample size of 895 per arm (1,790 women in total). After adjustment by 10% for loss to follow-up, protocol violations, and withdrawals, our planned total sample size of was 1,969 women, which we rounded to 1,970.

We planned to do an intention-to-treat analysis of all women randomly assigned who had a minilaparotomy procedure. All participants received the treatment to which they were allocated (e.g., participants randomized to have their minilaparotomy conducted by a CO actually had their procedure done by a CO, and vice versa). We included available data for all outcomes for participants who withdrew or were discontinued through the time their study participation ended. Observations with missing outcome data were not considered in the analyses. No missing data were imputed.

We assessed the primary outcome using the 95% confidence interval (CI) for the difference and the ratio between the proportion of participants with a major AE in the CO versus the AMO group. We used ordinal logistic regression, including adjustment for covariates (i.e., age, minilaparotomy type, education level, etc.) for the primary outcome analysis. We used a chi-squared test to assess the difference between the major AE rates for COs versus AMOs.

We assessed secondary outcomes as follows. To compare the safety of minilaparotomy provided by COs versus AMOs at different time points (intraoperatively, immediately postoperative, and at each follow-up visit), we compared the proportion of participants with major and minor AEs using a chi-squared test. We assessed variables related to performance of minilaparotomy between COs and AMOs as follows: procedure time and maximum reported pain experienced by the participant during the procedure between the 2 groups were compared using independent samples *t* tests; and requests for verbal instruction from the supervisor due to difficulty performing the minilaparotomy, requests for the supervisor to assist with the minilaparotomy, and inability to complete the minilaparotomy between the 2 groups were compared using chi-squared tests. We analyzed data on participant satisfaction using ordinal logistic regression and reported qualitative data on what participants liked about the minilaparotomy procedure, what they did not like, and if they would recommend it to a friend or family member, including reasons why.

We assessed self-efficacy of minilaparotomy providers based on 3 measures:

- A 10-item self-efficacy scale, with a range from 10 to 40, with higher values indicating greater self-efficacy, adapted from the General Self-Efficacy Scale^26^
- A measure of confidence, with a range between 3 and 12, with higher numbers indicating greater levels of confidence with the procedure
- A measure of comfort, using a scale from 3 to 12, with higher numbers indicating greater comfort with performing minilaparotomy

We used independent samples *t* tests to compare the outcomes between the 2 groups for each of the 3 measures. We used Stata version 13.1 for all analyses.

The 3-member Data and Safety Monitoring Board (DSMB) met twice during the trial. The DSMB reviewed 1 planned interim analysis after approximately one-third of the sample had their minilaparotomies and had completed their 7-day follow-up visit. They reviewed the proportion of participants with events and the number of participants recruited unmasked by treatment group and advised that the trial should continue until its planned completion.

The trial protocol was previously published^25^ and the trial is registered with ClinicalTrials.gov, Identifier NCT02944149, registered October 14, 2016.

## RESULTS

**FIGURE 1:**
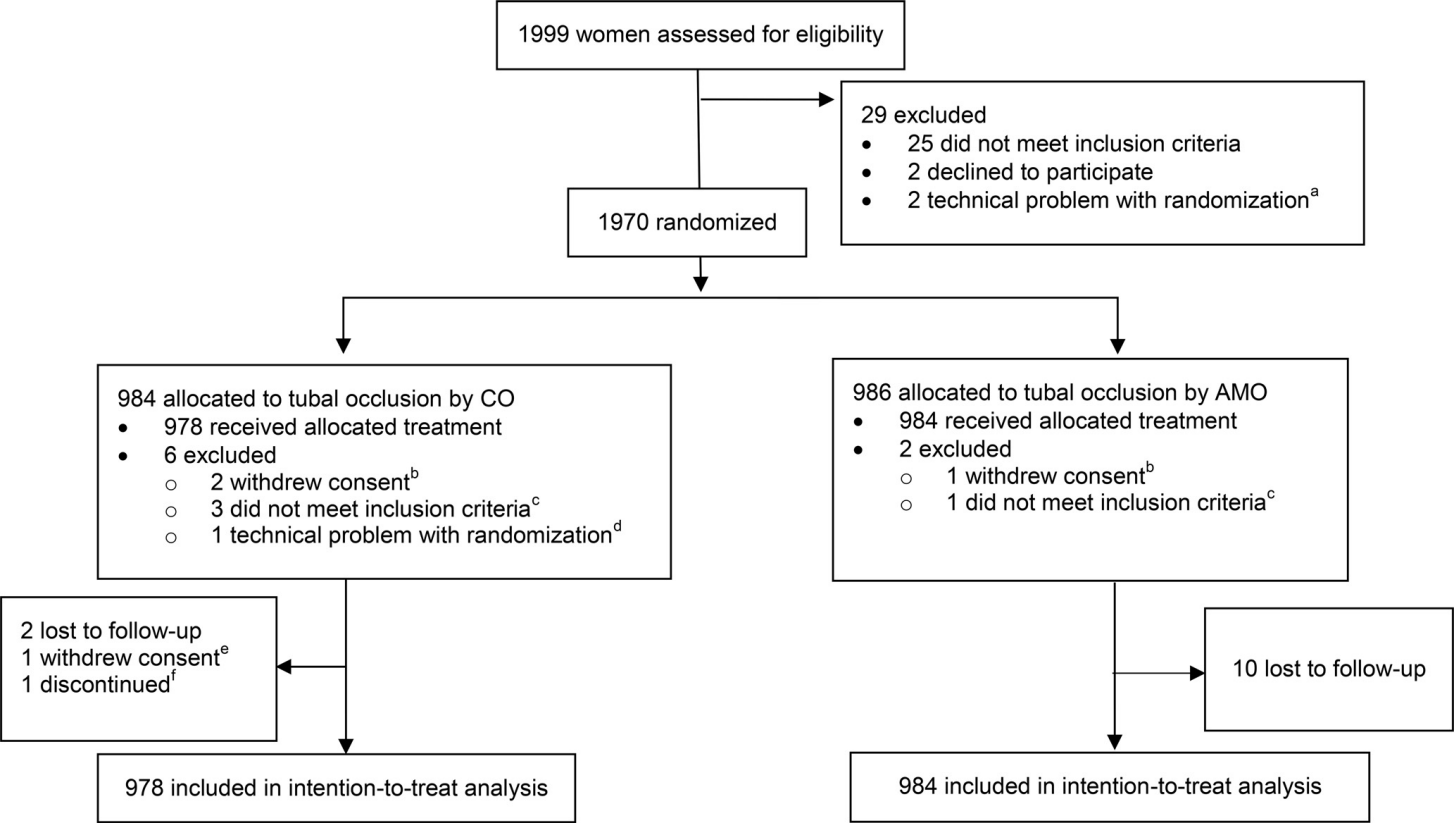
Trial Profile Abbreviations: AMO, assistant medical officers; CO, clinical officers. ^a^ Randomization was done via a text message service. In these 2 cases, a cellular network outage prevented the study site from randomizing the participants. ^b^ Just before the start of the procedure, 3 participants became nervous and withdrew consent. ^c^ These participants were deemed to have met the study eligibility criteria and were randomized. However, before the procedure commenced, it was decided that they did not meet the criteria for the following reasons: anemia, high blood pressure, pelvic inflammatory disease, or unexplained vaginal bleeding. ^d^ In this case, the participant was randomized, but a cellular network outage prevented the study site from determining the assigned random allocation group before the minilaparotomy procedure needed to be conducted for logistical reasons. The participant was discontinued. ^e^ The procedure was not completed because the participant was unsettled, as the procedure was taking a long time. She asked that they stop the procedure. ^f^ Adhesions made delivering the right tube a problem, and the procedure could not be completed, even with the supervisor's assistance.

Between December 6, 2016, and June 16, 2017, we assessed 1,999 women for eligibility, randomly allocating 1,970 (98.6%) women to minilaparotomy by a CO (n=984; 49.9%) or by an AMO (n=986, 50.1%) (Figure 1). A total of 8 participants—6 (0.6%) in the CO group and 2 (0.2%) in the AMO group—were excluded from the analysis because they did not have a minilaparotomy procedure: 4 were determined not to have met the inclusion and exclusion criteria after randomization but before the procedure, 3 withdrew consent after randomization but before the procedure, and in 1 instance there was a technical problem with the text-based randomization.

Over a 6-month period, we recruited 1,970 women and randomly allocated them to minilaparotomy by a clinical officer or by an assistant medical officer.

We analyzed data from 1,962 participants—978 (49.8%) in the CO group and 984 (50.2%) in the AMO group. The minilaparotomy procedure was started but not completed among 2 (0.2%) participants in the CO group. One participant withdrew her consent during the procedure, as it was taking a long time and she became unsettled. In the other case, it was not possible to deliver the right fallopian tube due to adhesions, even with the assistance of the supervisor. The procedure was not completed and the participant was discontinued. A total of 12 (0.6%) participants were lost to follow-up, 2 (16.7%) in the CO group and 10 (83.3%) in the AMO group. One of these participants attended her 3-day follow-up visit but did not return for any additional visits, while the other 11 participants were lost after making their 7-day visit. Available data from participants who withdrew, were discontinued, or were lost to follow-up were included in the analyses.

The 1,962 participants were distributed among the study sites as follows: 117 at Daraja Mbili Health Centre, 324 at Kaloleni Health Centre, 277 at Karatu Designated District Hospital, 447 at Levolosi Urban Health Centre, 83 at Longido Health Centre, 396 at Monduli District Hospital, and 318 at Mto wa Mbu Health Centre. The majority of participants were recruited during outreach services (1,715; 87.4%), as opposed to at the main study sites (247; 12.6%). The median number of minilaparotomy procedures conducted by an individual provider was 162, with a range of 20 to 256. The median (range) number of procedures conducted by COs and AMOs were similar, 161 (20, 238) and 162 (37, 256), respectively.

The majority of participants were recruited during outreach services.

**FIGURE 2:**
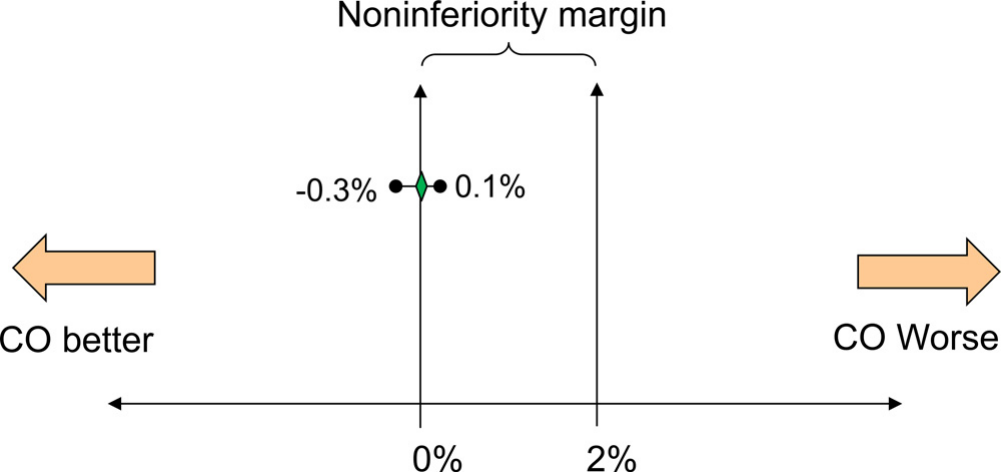
Interpretation of Risk Difference Between AMOs and COs for the Percentage of Women Experiencing a Major Adverse Event Abbreviations: AMO, assistant medical officer; CI, confidence interval; CO, clinical officer. The green diamond represents the point estimate of the risk difference (–0.1%) and the horizontal line to the left and right of the diamond represents the associated 2-sided 95% CI (–0.3%, 0.1%). Noninferiority of minilaparotomy performed by a CO is accepted because the upper limit of the 95% CI falls below the predefined noninferiority margin of 2%.

Baseline sociodemographic data, obstetric histories, family planning use, and reproductive intentions were similar between participants randomized to the 2 groups (Table 2 and Table 3). We noted no significant difference in the proportion of participants having a major AE following tubal occlusion by minilaparotomy between the 2 groups (CO group 0 [0.0%] of 978; AMO group 1 [0.1%] of 984) (Table 4). The risk difference for the percentage of women experiencing a major AE was –0.1% (95% CI: –0.3% to 0.1%). Because the upper limit of the 95% CI for the incidence rate difference fell below the predefined noninferiority margin (2%), the results show that tubal occlusion by minilaparotomy conducted by a CO is noninferior to tubal occlusion by minilaparotomy conducted by an AMO (Figure 2).

**TABLE 2.** Baseline Sociodemographic Characteristics of Minilaparotomy Participants, by Type of Service Provider Performing the Procedure

Characteristic	Clinical Officer (N=978)	Assistant Medical Officer (N=984)	Total (N=1962)
**Age groups, years, No. (%)**			
18–24	2 (0.2)	2 (0.2)	4 (0.2)
25–30	38 (3.9)	34 (3.5)	72 (3.7)
31–35	149 (15.2)	140 (14.2)	289 (14.7)
36–40	526 (53.8)	514 (52.2)	1040 (53.0)
41–45	249 (25.5)	276 (28.1)	525 (26.8)
46–50	14 (1.4)	18 (1.8)	32 (1.6)
**Age, years, mean (SD [range])**	37.8 (3.9 [21–50])	37.9 (3.7 [22–50])	37.9 (3.8 [21–50])
**Marital status, No. (%)**			
Married/cohabitating	922 (94.3)	933 (94.8)	1855 (94.6)
Divorced/separated	32 (3.3)	32 (3.3)	64 (3.3)
Widowed	18 (1.8)	15 (1.5)	33 (1.7)
Single	6 (0.6)	4 (0.4)	10 (0.5)
**Education level, No. (%)**			
None	73 (7.5)	64 (6.5)	137 (7.0)
Some primary	113 (11.6)	117 (11.9)	230 (11.7)
Completed primary	713 (72.9)	721 (73.3)	1,434 (73.1)
Some secondary	37 (3.8)	34 (3.5)	71 (3.6)
Completed secondary	39 (4.0)	43 (4.4)	82 (4.2)
Post-secondary	3 (0.3)	5 (0.5)	8 (0.4)
**Religion, No. (%)**			
Lutheran	350 (35.8)	365 (37.2)	715 (36.5)
Catholic	274 (28.0)	282 (28.7)	556 (28.3)
Muslim	198 (20.3)	179 (18.2)	377 (19.2)
Protestant	96 (9.8)	109 (11.1)	205 (10.4)
Other	60 (6.1)	49 (5.0)	109 (5.6)
**Occupation, No. (%)**			
Farmer	711 (72.7)	674 (68.5)	1385 (70.6)
Small-scale business	183 (18.7)	209 (21.2)	392 (20.0)
Housewife	30 (3.1)	56 (5.7)	86 (4.4)
Teacher	17 (1.7)	18 (1.8)	35 (1.8)
Other	29 (3.0)	18 (1.8)	47 (2.4)
Missing	8 (0.8)	9 (0.9)	17 (0.9)

**TABLE 3.** Baseline Measures of Obstetric History, Family Planning Use, and Reproductive Intentions of Minilaparotomy Participants, by Type of Service Provider Performing the Procedure

Characteristic	Clinical Officer (N=978)	Assistant Medical Officer (N=984)	Total (N=1962)
**Ever pregnant, No. (%)**	978 (100.0)	984 (100.0)	1962 (100.0)
**Outcome of pregnancies, No. (SD)**			
Live birth	5.8 (1.6)	5.9 (1.6)	5.8 (1.6)
Stillbirth	0.02 (0.2)	0.02 (0.2)	0.02 (0.2)
Miscarriage/abortion	0.3 (0.6)	0.2 (0.6)	0.3 (0.6)
**No. of living children, No. (SD)**			
Boys	3.0 (1.3)	3.0 (1.3)	3.0 (1.3)
Girls	2.8 (1.3)	2.9 (1.3)	2.8 (1.3)
Total	5.7 (1.5)	5.9 (1.6)	5.8 (1.6)
**Last family planning method used, No. (%)**			
Injectables	371 (37.9)	402 (40.9)	773 (39.4)
Implant	221 (22.6)	211 (21.4)	432 (22.0)
Oral contraceptives	215 (22.0)	210 (21.3)	425 (21.7)
Intrauterine device	43 (4.4)	46 (4.7)	89 (4.5)
Male condom	27 (2.8)	22 (2.2)	49 (2.5)
Periodic abstinence	4 (0.4)	10 (1.0)	14 (0.7)
Withdrawal	8 (0.8)	6 (0.6)	14 (0.7)
Lactational Amenorrhea Method	2 (0.2)	0 (0.0)	2 (0.1)
None	87 (8.9)	77 (7.8)	164 (8.4)
**First heard about female sterilization from, No. (%)**			
Health care provider	840 (85.9)	860 (87.4)	1,700 (86.7)
Other sterilized person	53 (5.4)	38 (3.9)	91 (4.6)
Friend or relative	50 (5.1)	40 (4.1)	90 (4.6)
Spouse	19 (1.9)	22 (2.2)	41 (2.1)
Community leader	4 (0.4)	11 (1.1)	15 (0.8)
Public outreach worker	3 (0.3)	7 (0.7)	10 (0.5)
Brochure	3 (0.3)	3 (0.3)	6 (0.3)
Poster	3 (0.3)	2 (0.2)	5 (0.3)
Radio	2 (0.2)	0 (0.0)	2 (0.1)
TV	1 (0.1)	1 (0.1)	2 (0.1)
**Main reason for wanting female sterilization, No. (%)**			
Desired family size completed	850 (86.9)	877 (89.1)	1,727 (88.0)
Financial/economic reasons	72 (7.4)	51 (5.2)	123 (6.3)
Health reasons	29 (3.0)	36 (3.7)	65 (3.3)
Complications from a previous birth	18 (1.8)	15 (1.5)	33 (1.7)
Encouraged by family, friend, or spouse	8 (0.8)	5 (0.5)	13 (0.6)
Single mother with a disabled child	1 (0.1)	0 (0)	1 (0.1)
**Time since deciding not to have any more children, years, mean (SD [range])**	1.9 (2.1 [0.003,^a^ 26])	1.9 (2.0 [0.003,^a^ 20])	1.9 (2.1 [0.003,^a^ 26])

a 0.003 years=1 day.

**TABLE 4.** Primary and Secondary Outcomes, by Type of Service Provider Performing the Procedure

Outcome	Clinical Officer	Assistant Medical Officer	OR (95% CI)	*P* Value
**Primary outcome**				
Major AEs, n/N (%)	0/978 (0.0)	1/984 (0.1)	0.0005 (0.00007, 0.0036)	.32
**Secondary outcomes**				
*Major and minor AEs at different time points during the study, n/N (%)*				
Intraoperatively	0/978 (0.0)	0/984 (0.0)	NA	NA
Immediately postoperative	0/978 (0.0)	0/984 (0.0)	NA	NA
3 days postoperative	1/969 (0.1)	0/976 (0.0)	0.0005 (0.000072, 0.0036)	.32
7 days postoperative	2/976 (0.2)	3/975 (0.3)	1.5 (0.3, 8.9)	.66
Unscheduled postoperative visits^a^	1/4 (25.0)	3/13 (23.1)	0.80 (0.2, 3.0)	.94
*Performance of tubal occlusion by minilaparotomy*				
Time to complete procedure, minutes, mean (SD [range])	26.0 (1.0 [14, 65])	26.0 (1.0 [15, 90)	NA	.42
Requested verbal instruction from the supervisor due to difficulty performing the procedure,^b^ n/N (%)	15/978 (1.5)	20/984 (2.0)	0.75 (0.36, 1.56)	.40
Requested the supervisor assist with the procedure,^c^ n/N (%)	14/978 (1.4)	13/984 (1.3)	1.08 (0.47, 2.52)	.80
Inability to complete procedure,^d^ n/N (%)	2/978 (0.2)	0/984 (0.0)	NA	.25
Maximum pain during procedure,^e^ mean (SD)	4.12 (2.4)	4.11 (2.4)	NA	.98
Participant very satisfied with minilaparotomy, n/N (%)	834/969 (86.1)	831/976 (85.1)	1.01 (0.88, 1.15)	.34
*Self-efficacy of providers in performing minilaparotomy*,^f^ *mean (SD)*				
General self-efficacy	32.3 (6.1)	31.3 (7.5)	NA	.79
Confidence	10.9 (0.9)	11.5 (0.8)	NA	.21
Comfort	11.4 (0.5)	10.9 (1.7)	NA	.41

Abbreviations: AE, adverse event; CI, confidence interval; NA, not applicable; OR, odds ratio; SD, standard deviation.

a All AEs observed during unscheduled visits occurred between Days 2 and 6 postoperatively.

b Most of these cases (n=21; 60.0%) involved difficulty locating or delivering the fallopian tube(s) due to obesity, adhesions, or unspecified reasons. Other reasons included unsettled/restless participant, abnormal uterus, difficulty placing the uterine elevator, and difficulty finding the uterus after the incision was made.

c These cases are a subset of those where verbal instruction was requested by the provider.

d In 1 case, the participant was unsettled because the procedure was taking a long time. She asked that they stop. In the other case, adhesions made delivering the right fallopian tube a problem. It was not possible to complete the procedure.

e 0=no pain, 10=worst pain possible.

f General self-efficacy scale: 10=lower self-efficacy, 40=higher; confidence and comfort scales: 3=lower confidence or comfort, 12=higher.

There was no significant difference in the proportion of participants with a major adverse event following tubal occlusion by minilaparotomy between the 2 group.

We noted no significant differences between the 2 treatment groups in any of the secondary outcomes (Table 4). There were no differences in rates of AEs (major and minor combined) at any time during the procedure or follow-up period. Measures of performance did not differ between groups, including mean procedure time, requests for verbal instruction from the supervisor or for the supervisor to assist with procedures due to difficulty performing procedures, inability to complete procedures, reported pain during procedures, and participant satisfaction. Measures of provider self-efficacy did not differ between the 2 groups and all 14 providers said they were interested in continuing to perform minilaparotomy after the study.

Ten (0.5%) AEs occurred among 9 participants (1 participant had 2 concurrent AEs). Similar numbers of AEs (COs 4 [0.4%]; AMOs, 6 [0.6%]) were seen in both treatment groups (risk difference: –0.2% [95% CI: –0.8% to 0.4%]). All AEs occurred during follow-up. One AE was classified as major, a serious wound infection that occurred 4 days after a procedure done by an AMO. The wound was opened and drained, and the participant was hospitalized for close monitoring and to receive injectable antibiotics. She healed as expected, with no sequelae. The minor AEs included 4 (0.2%) wound infections, 3 (0.2%) cases of abdominal pain 6–7 days post-procedure requiring oral pain relievers, 1 (0.1%) case of wound dehiscence, and 1 (0.1%) case of nausea and vomiting. All the minor AEs were resolved with conservative management and without any sequelae.

The majority of the minilaparotomy procedures performed were interval (n=1,901; 96.9%), with few postpartum (n=58; 3.0%) and postabortion (n=3; 0.2%) procedures. We noted no significant differences between the treatment groups in variables related to performance of the minilaparotomy procedures (Table 4 and Table 5). There were few cases overall where the provider reported requesting verbal instruction from the supervisor due to difficulty with the procedure (35; 1.8%). The provider requested the supervisor assist during the procedure in 27 of those cases (1.4% of all procedures). Most of the difficult cases (n=21; 60.0%) involved difficulty in locating or delivering the fallopian tube(s) due to obesity, adhesions, or unspecified reasons. This was also the most common reason why a provider requested that a supervisor assist with the procedure (n=18; 66.7%).

**TABLE 5.** Additional Performance Measures, by Type of Service Provider Performing the Minilaparotomy Procedure

	Clinical Officer (N=978)	Assistant Medical Officer (N=984)	Total (N=1962)	*P* Value
Additional local anesthesia injected during procedure, No. (%)	5 (0.5)	4 (0.4)	9 (0.5)	.75
Change of anesthesia to general or spinal, No. (%)	0 (0.0)	0 (0.0)	0 (0.0)	NA
Estimated incision length 2–3 cm, No. (%)	978 (100.0)	984 (100.0)	1962 (100.0)	NA
Extension of abdominal incision needed, No. (%)	0 (0.0)	1 (0.1)	1 (0.1)	1.0
Switch to laparotomy, No. (%)	0 (0.0)	0 (0.0)	0 (0.0)	NA
Discharged well from facility on day of procedure, No. (%)	978 (100.0)	984 (100.0)	1962 (100.0)	NA

At the Day 3 follow-up visit, 1,665 (85.6%) participants said that they were very satisfied and 253 (13.0%) said they were somewhat satisfied with the provider who had performed their procedure. There were no significant differences between the treatment groups (very satisfied *P*=.34; somewhat satisfied *P*=.90). At the Day 42 visit, 1,938 (99.5%) said they would recommend minilaparotomy to a friend or family member, with no significant difference between treatment groups (*P*=.75). When participants were asked what they liked about the minilaparotomy at the 42-day visit, top responses included (multiple responses were possible): that healing went well (n=1,301; 66.8%), everyone at the facility was nice (n=840; 43.1%), the procedure was quick (n=815; 41.8%), and they experienced less pain than expected during and after the procedure (n=658; 33.8%). Twenty participants (n=10; 1.0% in each group) said that there was nothing they liked about the procedure. When we asked participants what they disliked, 1,829 (93.9%) participants reported that there was nothing they disliked, with a few participants (1.5% or less) reporting that they experienced more pain than expected or that the procedure took a long time, among other reasons.

## DISCUSSION

Our results show that tubal occlusion by minilaparotomy can be conducted safely and effectively by trained COs, with no evidence of increased risk of major or minor AEs associated with the procedure, problems with performance of the procedure, or negative effects on satisfaction among women undergoing the procedure, compared with procedures performed by an AMO. Although systematic reviews of older studies^19^^,^^20^ and results from more recent nonrandomized studies^21^^–^^23^ provide some evidence to support provision of minilaparotomy by nonphysicians, the results of our large, multicenter randomized trial provide solid empirical evidence to support changing international guidelines and country-level regulations to allow task shifting of minilaparotomy to trained COs and similar nonphysician cadres.

**Figure:**
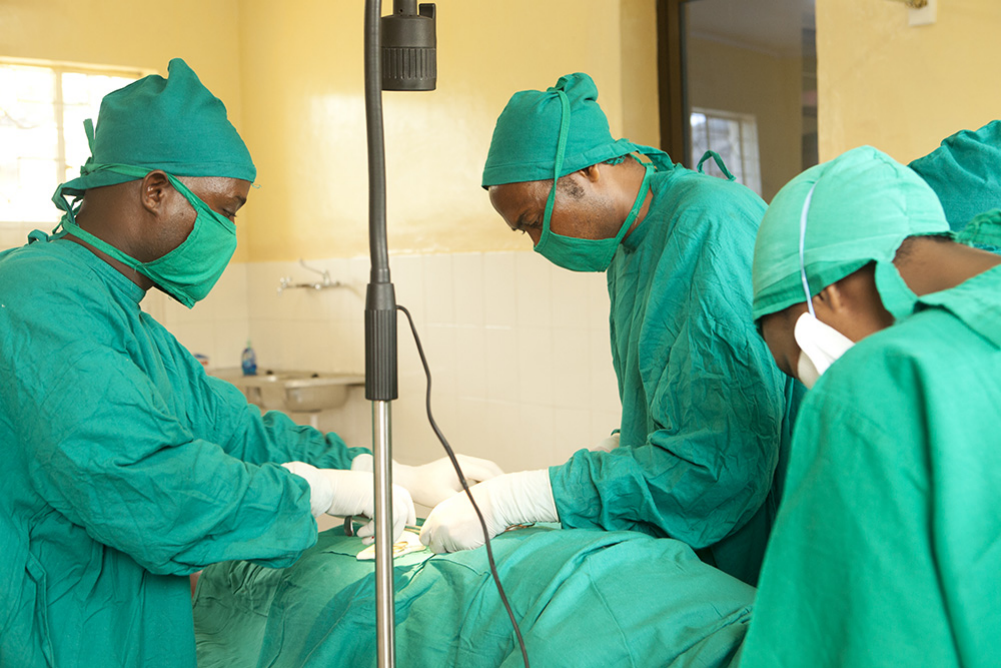
A woman undergoes tubal ligation by minilaparotomy in a health facility in northern Tanzania. © 2015 EngenderHealth/S. Lewis

Tubal occlusion by minilaparotomy can be conducted safely and effectively by trained clinical officers.

The terminology describing nonphysician clinicians varies from country to country, which may make it difficult to interpret task shifting studies. WHO uses “associate clinician” to refer to nonphysicians who generally have 3–4 years of postsecondary training in diagnosis and management of common medical and surgical conditions.^18^ This is the case with COs in Tanzania, who have undergone 3 years of specialized training, and some other African countries (e.g., Kenya, Uganda, and Zambia), although in others (e.g., Malawi), similar named cadres have more postsecondary training. In our study, most of the providers had some prior surgical experience, although the COs' experience was restricted to minor surgical procedures such as wound repair, draining of abscesses, and male circumcision, whereas the AMOs had experience with more complex surgeries such as cesarean deliveries and appendectomy. Nonetheless, all the COs successfully completed the minilaparotomy training, safely conducted procedures during the study, and expressed comfort and confidence in terms of the self-efficacy measure, irrespective of prior surgical experience.

Quality of the training for task shifting minilaparotomy may be more important than the trainees' prior surgical experience. It is critical that minilaparotomy training focus on careful presurgical screening and good surgical technique in order to reduce the risk of intra- and postoperative complications, as well as detecting and dealing with possible complications such as injuries to the viscera, bleeding from the procedure site, and adverse drug reactions. This includes emergency preparedness and ensuring that trainees understand when to seek assistance or refer a client.

Inadequate numbers of trained health care workers due to shortages, inequitable geographic distribution, and difficulties recruiting and retaining trained health workers was identified as a key reason the health-related Millennium Development Goals were not achieved in many countries.^27^^,^^28^ Physicians tend to be concentrated in urban areas, even though the majority of the population in many resource-limited settings resides in rural areas. They also end up having to prioritize curative services or higher-level tasks, leaving less time for preventive services such as family planning. These human resource constraints are likely to have a more significant impact on access to clinic-based family planning methods such as minilaparotomy.^18^

In addition to reducing unintended pregnancies, satisfying unmet need for contraception reduces the numbers of induced abortions, provides substantial health benefits (including reducing maternal, newborn, and child morbidity and mortality), and contributes to a host of other development objectives necessary to achieving the Sustainable Development Goals (SDGs).^29^ Expanding the health workforce will be critical to improving health, strengthening health systems, and making progress toward the SDGs. The WHO High-Level Commission on Health Employment and Economic Growth's recommendations state that task shifting, among other approaches that make optimal use of the available workforce, should be urgently pursued,^30^ a recommendation seconded by the *Lancet* Commission on the future of health in sub-Saharan Africa.^31^

The only other randomized study to explore task shifting tubal occlusion by minilaparotomy to nonphysicians was conducted among 292 women undergoing postpartum minilaparotomy in a large urban hospital in Thailand, with the procedure conducted by nurse-midwives or doctors.^32^ No differences in AE rates were seen, although (unlike our results) they reported that nurse-midwives took significantly longer to conduct the procedure; the time difference was relatively short (approximately 7 minutes) and may be outweighed by the advantages of having nurse-midwives provide the procedure. Unlike the study in Thailand, for logistical reasons and to ensure adequate recruitment of study participants in the time we had to carry out our study, we conducted most of the minilaparotomy procedures during outreach services. The overall rate of major AEs we observed was low and comparable to rates reported by others when minilaparotomy was provided by COs in both clinic and outreach settings, although it can be difficult to compare AE rates across studies, given different definitions (there is no universally accepted system for defining AEs/complications) and approaches to recording their occurrence.^21^^–^^23^

Our data show that minilaparotomy can be safely and effectively provided in outreach settings, whether by COs or AMOs, and supports the use of this approach to expand access to minilaparotomy. Outreach services are commonly used to increase access to a range of family planning and other health services in remote, rural, and underserved areas in Tanzania and many other developing regions. National guidelines for family planning outreach activities typically include details on how such services should be planned (including arrangements for referral and transport of clients in case of emergency), implemented (including having necessary drugs and equipment on hand), and monitored (including a supervisory team to provide quality assurance and back-up support when needed), to ensure that services are safe. Additionally, outreach teams usually include highly qualified providers, with extensive experience and expertise necessary to both reduce the risk of emergencies and handle them should they arise.

Our study also supports the use of outreach services to expand access to minilaparotomy.

We saw no evidence of differences in other outcomes between the 2 groups that would raise concern about COs conducting minilaparotomy and no evidence that provision of minilaparotomy by COs was any less acceptable to women than when provided by AMOs. The high acceptability of minilaparotomy provided by COs has also been reported by others.^21^^–^^23^ We found high and equal levels of general self-efficacy, as well as confidence and comfort in performing minilaparotomy, among both COs and AMOs. All of the providers said they would be interested in continuing to conduct minilaparotomy, although the COs are unable to do so without a change in the Tanzanian government guidelines. Our data also demonstrate what appears to be high demand for female sterilization services in Tanzania, given the large number of women we were able to recruit for the study in a relatively short period of time.

There appears to be high demand for female sterilization in Tanzania, given the large number of women recruited for the study in a relatively short amount of time.

### Limitations

One limitation of our study is that it was not masked. In view of the nature of the intervention and the way in which services are provided in Tanzania, it was not practical to mask study or facility staff (or the women themselves) to the treatment group (i.e., we could not hide which type of provider was doing the procedure) or to have the outcome assessments done by a provider unaware of the treatment allocation. Another limitation is that the duration of follow-up that was practical for our study was insufficient to determine whether efficacy in preventing pregnancy was similar between the 2 provider groups. We were unable to include other nonphysician cadres conducting minilaparotomy in the study. There is some evidence—primarily from Asia—suggesting that trained nurses and nurse-midwives can safely and effectively provide minilaparotomy, although the available evidence is limited and weak.^19^^,^^20^ This issue deserves further exploration as an additional way to increase access.

## CONCLUSION

Our results demonstrate that task shifting of tubal occlusion by minilaparotomy to COs is safe, effective, and acceptable to women. These results provide the evidence needed to support policy change at the national level in Tanzania and beyond, helping to meet the rising demand for female sterilization among women who wish to limit their childbearing and improving family planning method mix.^4^ Increasing the voluntary use of modern family planning methods, including permanent methods such as female sterilization, will play a critical role in meeting women's reproductive intentions and improving maternal, neonatal, and child morbidity and mortality, and will be vital to increasing the contraceptive prevalence rates in developing regions critical to achieving the SDGs.^29^

The results from this trial provide the evidence needed to support policy change at the national level in Tanzania and beyond.

## Notes

**Peer Reviewed**

**Funding:** This study was funded by the generous support of the American people through the United States Agency for International Development (USAID) under the term of the Associate Cooperative Agreement No. 621-LA-13-00001 through LWA No. GPO-A-00-08-00007-00 to EngenderHealth.

**First Published Online:** August 16, 2018
